# DES c.1360C>T: A Rare Desmin Variant Causing Early Distal Myopathy and Cardiomyopathy

**DOI:** 10.7759/cureus.36368

**Published:** 2023-03-19

**Authors:** Rafael Dias, Teresa C Aguiar

**Affiliations:** 1 Neurology, Hospital Central do Funchal, Funchal, PRT

**Keywords:** (p.(arg454trp)), c.1360c>t, cardiomyopathy, des, desmin-related myopathy, desmin

## Abstract

Desmin-related myopathies are characterized by progressive, distal skeletal muscle weakness, cardiomyopathy, and cardiac conduction disease caused by mutations of Desmin. A 43-year-old man with a history of heart transplant due to heart failure associated with restrictive cardiomyopathy, presented with slowly progressive distal muscle weakness in all four extremities for five years. On examination, predominantly distal quadriparesis and atrophy were noted, worse on the upper limbs, and reduced reflexes with normal sensation. His electromyographic studies were suggestive of subacute moderate motor axonal polyneuropathy secondary to the transplant immunosuppression. The patient’s father died at 33 years due to heart failure, and his 37-year-old brother, who also had a heart transplant, had noticed the development of muscle atrophy. Another electroneuromyography performed on our index patient confirmed features consistent with a distal myopathy. A genetic panel for distal myopathies with cardiac involvement identified the pathological desmin gene mutation DES (NM_001927.4) - c.1360C>T; (p.(Arg454Trp)). Desmin-related myopathies are a diagnostic challenge. The beginning of neurological symptoms several years after the cardiac symptoms and the use of immunosuppressive agents may have contributed to the early misdiagnosis.

## Introduction

Myofibrillar myopathies (MFM) compose a group of inherited heterogeneous diseases with similar histopathological patterns characterized by protein aggregates and myofibrillar disintegration [1]. They are usually inherited through an autosomal dominant pattern; however, autosomal recessive and X-linked transmission have been described. [2].

Several gene variants have been linked to the development of MFM including desmin (*DES*), α-B-crystallin (*CRYAB*), myotilin (*MYOT*), LIM-domain-binding 3/Z band alternatively spliced PDZ-containing protein (*LDB3/ZASP*), filamin C (*FLNC*), and Bcl2-associated athanogene-3 (*BAG3*), and several others related to atypical clinical presentations [1].

Desmin, a protein encoded by the *DES *gene, is an intermediate filament in cardiac, skeletal, and smooth muscle cells, playing a central role in the structural maintenance of the muscle cell [3]. Desmin-related myopathies (DRM) are a type of MFM generally characterized by progressive, distal skeletal muscle weakness, cardiomyopathy, and cardiac conduction disease [4]. More than 50 mutations of the *DES *gene have been described, with most mutations being missense; however, null or deletions are also possible and the clinical presentation is highly variable, with no specific genotype-phenotype correlation being detected [3].

Despite the variable presentation of different DRM and their usual age of presentation, Oomen et al. described a rare desmin variant (*DES*, c.1360C>T; (p.(Arg454Trp))) causing penetrant life-threatening arrhythmic cardiomyopathy early in life [4]. This mutation has only been described in 10 cases in the literature and appeared to be associated with earlier symptom onset, usually before adulthood, and a very severe cardiac phenotype and high penetrance, needing a pacemaker or implantable cardioverter-defibrillator (ICD) at an earlier age; however, the neurological symptoms remained underdescribed [4].

This specific mutation may even be underdiagnosed and under-published due to its high association with cardiac disease and fatal arrhythmias. The presenting article describes two more confirmed cases of the same family of this highly penetrant *DES *variant, focusing on the neurological aspects of the disease and the pitfalls that can delay the diagnosis. This article was previously presented as a meeting abstract at the Fifth Congress of the European Academy of Neurology in Oslo, Norway on June 29, 2019.

## Case presentation

**Figure 1:**
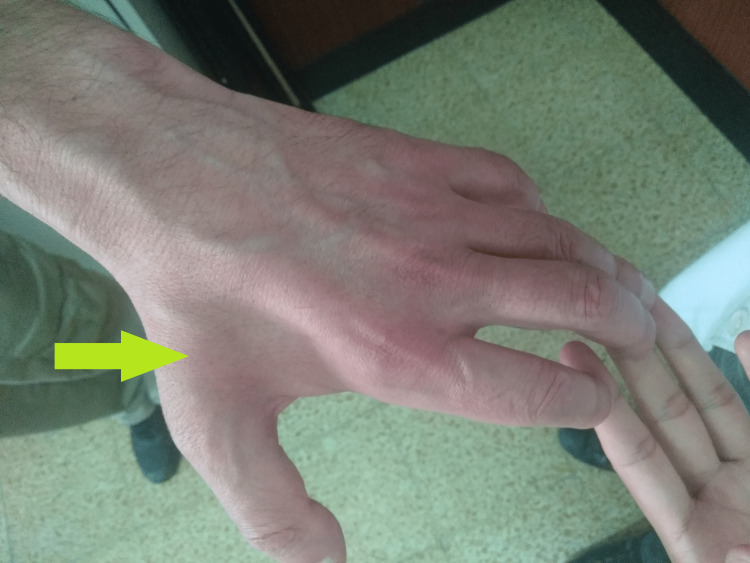
Patient's left hand Arrow marks atrophy of the interosseous muscles

**Figure 2:**
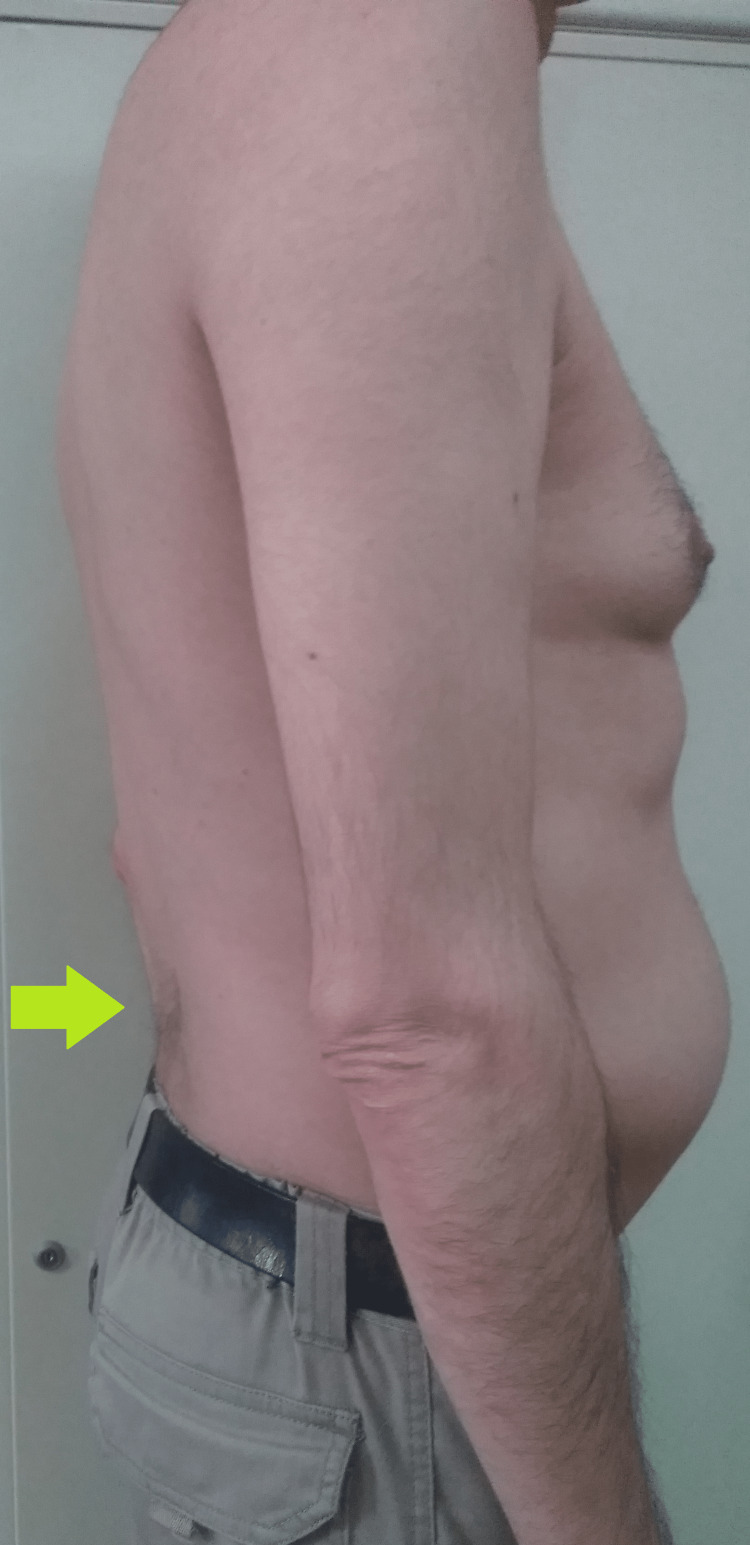
Patient's trunk, lateral view Arrow marks loss of normal spinal curvature

A 43-year-old man with a history of a heart transplant, came to a neurology consult due to complaints of muscular weakness and atrophy with five years of progression. The patient presented with quadriparesis with predominant involvement of upper extremities associated with atrophy of interosseous muscle of the hands (Figure 1), loss of normal spinal curvature (Figure 2), and a bilateral foot drop with radial and ankle jerk reflexes abolished.

Regarding personal history, the patient reported episodes of transient sudden loss of consciousness starting at the age of 17, eventually being diagnosed with an unspecified cardiac conduction disease and having a pacemaker implanted at the age of 27. Later, at the age of 32, due to the development of severely restricted cardiomyopathy and heart failure, he underwent heart transplantation and was initiated on tacrolimus as immunosuppressive therapy.

When the neurological symptoms were first noticed at the age of 38, the patient presented with distal muscle weakness of the upper limbs and the lower limbs with no effect on daily activities. An electroneuromyography (ENMG) was performed suggestive of a subacute moderate motor axonal polyneuropathy, at the time thought to be related to the heart transplant immunosuppression. The patient suspended tacrolimus and was started on cyclosporin; however, there was no improvement in the symptoms and the patient got progressively worse until he was sent for a neurology evaluation.

**Figure 3:**
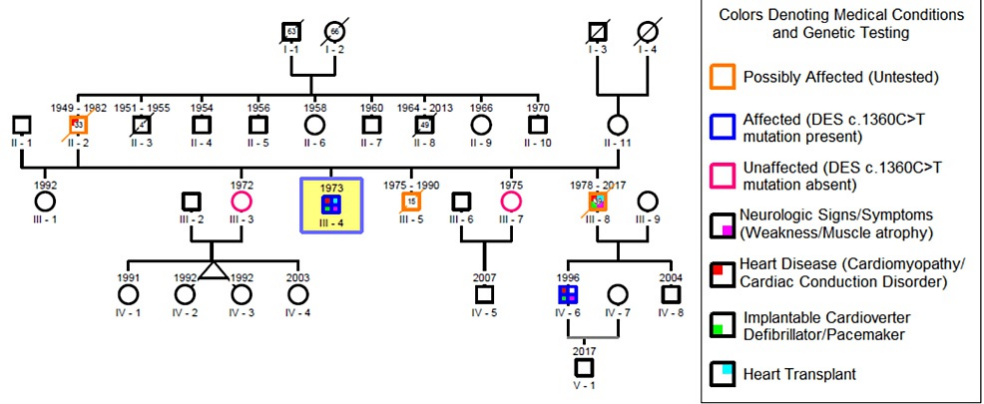
Family genogram of patient III-4, who is marked as index patient

Family history was remarkable (Figure 3). The patient’s father (II-2) had a pacemaker implanted at the age of 28 and died at the age of 33 years due to heart failure after refusing heart transplantation. His 37-year-old brother (III-8) who also had a pacemaker implanted after several episodes of sudden loss of consciousness and a heart transplant at the age of 27 years due to heart failure, had noticed the development of muscle atrophy, mostly on the lower legs along with difficulty in breathing. Another brother (III-5) had died at the age of 15 of sudden cardiac arrest.

He also had a nephew (IV-6) with an ICD implanted at the age of 11 years after unspecified arrhythmias were detected on an electrocardiogram (EKG), and although he didn’t complain of muscle weakness, on neurological examination presented with atrophy of the temporalis muscle, interosseous muscles of the hands and gastrocnemius muscles, and a distal paresis of lower limb and hands classified as degree 4/5. The patient also had two healthy sisters (III-3 and III-7).

Considering the family history filled with early onset cardiac disease and the development of muscle atrophy and weakness in several family members, the ENMG was repeated showing a pattern suggestive of distal myopathy, although serum creatine kinase (CK) levels were normal. A next-generation sequencing panel with 72 genes for distal myopathies with cardiac involvement and Sanger sequencing validation for low qualities variables (GATK quality score) was conducted on the patient and the rare pathological desmin gene mutation *DES *(NM_001927.4) - c.1360C>T; (p.(Arg454Trp)) was identified.

Due to the dominant pattern of inheritance of this variant, all close family members were contacted, and clinically evaluated to look for signs or symptoms of a DRM and if symptomatic sent for testing. Unfortunately, by the time the mutation was identified in our patient (III-4), his brother (III-8) had died of respiratory failure associated with sepsis. He couldn’t be tested but our patient’s nephew (IV-6) tested positive for the same variant. Both sisters were negative (III-3 and III-7).

All positive family members and respective untested descendants were informed of the diagnosis and begin follow-up on cardiology and neurology consultations.

## Discussion

Myofibrillar myopathies and specifically desmin-related myopathies are a diagnostic challenge, mostly due to their rarity and variety of clinical presentations. In this case, two aspects may have contributed to the misdiagnosis: the first neurological symptoms being noticed several years after the cardiological ones and an early misleading ENMG that pointed to the immunosuppressive agents as a possible etiology.

Van Spaendonck-Zwarts et al. reported that most patients with DRM that presented with isolated neurological symptoms had a mutation on the 2B domain, while most of the patients with isolated cardiological symptoms had a mutation on the head or tail domain [2].

The variant *DES *c.1360C>T; (p.(Arg454Trp)) described is located in the carboxy “tail” domain [4]; however, as we could see with the patient’s nephew (IV-6), who didn’t complain of muscle weakness but had already signs on neurological examination, the neurological signs on our patient may have started long before they were first noticed, raising the question if this specific mutation has an early cardiac phenotype and a later neurological one or whether the cardiological signs are just more easily noticeable.

Another interesting aspect is the seeming preponderance of the *DES *c.1360C>T; (p.(Arg454Trp)). mutation in the male population. Of the 10 cases already reported, 70% are males and 30% are females [4]. In this family report, only males are affected (two confirmed, three probably affected), raising this difference further. Although no females in this family had the mutation, one could postulate that affected females may have lower penetrance of the disease. Indeed, although DRM is usually of autosomal dominant inheritance, the penetrance of the disease is variable, being possible to remain asymptomatic for life [3].

Females may have different skeletal muscle cell composition and gene activation. However, nonspecific to desmin, studies have already demonstrated several differences related to skeletal muscles between genders [5], and other DRM reviews noticed that males appeared to be more affected by cardiomyopathy than females [2].

DRM’s first symptoms generally appear in early adulthood [2,3,6], consisting of distal muscle weakness usually starting on the lower limbs, and gradually spreading to the upper limbs [2,6]. However, some cases may present as predominantly proximal myopathies, leading to considering limb-girdle dystrophy as a possible diagnosis [3]. Cardiac involvement is usually characterized by the development of cardiomyopathy, with no specific type (dilated, hypertrophied, restricted, arrhythmogenic) being related to one mutation [2,3].

The *DES *variant described here appears to have an earlier onset beginning before adulthood [4]. In our family, most affected members had episodes of sudden transient loss of consciousness beginning around adolescence and the patient IV-6 had already signs of neuromuscular disease.

Cardiac conduction disease can present with any type of arrhythmia or conduction block or even sudden cardiac arrest [2-4]. In the present family, although unconfirmed, patient III-5, who died of sudden cardiac arrest, may have had the variant and the presenting sign could have been the cardiac arrest; however, it’s impossible to confirm. Unfortunately, the EKGs performed weren’t registered electronically, and data couldn’t be accessed.

In DRMs, despite myopathic involvement, CK levels are usually normal or only slightly elevated and electroneuromyography generally shows myogenic traces; however, mixed or neurogenic traces can be present and may be misleading in the early stages of the disease [2,3].

Our patient's CK levels were normal despite the degree of quadriparesis noticed, and his first ENMG, in the context of immunosuppression after cardiac transplantation was interpreted as subacute moderate motor axonal polyneuropathy, which delayed diagnosis for almost five years. Perhaps a neurology evaluation at that time could have improved ENMG accuracy.

Imaging of DRM usually through computed tomography or magnetic resonance imaging shows early fatty degeneration of leg muscles, progressing to muscles of the thighs at a later stage [3]. Due to the present genetic diagnosis, a muscle image of the affected individuals wasn’t performed.

## Conclusions

Desmin-related myopathies are a rare inherited disease, and with a highly variable phenotype, a high degree of suspicion must be raised to diagnose these diseases early in life, not only to prevent sudden death but also to initiate muscular rehabilitation and physical therapy to improve quality of life. A DRM myopathy must always be looked for in patients with a history of cardiac disease that develop muscle weakness and atrophy.
